# Mediterranean Diet and Bladder Cancer Risk in Italy

**DOI:** 10.3390/nu10081061

**Published:** 2018-08-10

**Authors:** Francesca Bravi, Maria-Eleni Spei, Jerry Polesel, Matteo Di Maso, Maurizio Montella, Monica Ferraroni, Diego Serraino, Massimo Libra, Eva Negri, Carlo La Vecchia, Federica Turati

**Affiliations:** 1 Department of Clinical Sciences and Community Health, Università degli Studi di Milano, 20133 Milan, Italy; francesca.bravi@unimi.it (F.B.); matteo.dimaso@unimi.it (M.D.M.); monica.ferraroni@unimi.it (M.F.); federica.turati@unimi.it (F.T.); 2 Department of Hygiene, Epidemiology and Medical Statistics, School of Medicine, National and Kapodistrian University of Athens, 11527 Athens, Greece; marilena_0108@hotmail.com; 3 Unit of Cancer Epidemiology, IRCCS CRO Aviano National Cancer Institute, 33081 Aviano, Italy; polesel@cro.it (J.P.); serrainod@cro.it (D.S.); 4 Department of Public Health and Pediatric Sciences, Università degli Studi di Torino, CTO Hospital, 10126 Turin, Italy; 5 Unit of Epidemiology, Istituto Tumori Fondazione Pascale IRCCS, 80131 Naples, Italy; m.montella@istitutotumori.na.it; 6 Laboratory of Translational Oncology & Functional Genomics, Department of Biomedical and Biotechnological Sciences, Università di Catania, 95124 Catania, Italy; m.libra@unict.it; 7 Department of Biomedical and Clinical Sciences, Università degli Studi di Milano, 20157 Milan, Italy; eva.negri@unimi.it

**Keywords:** Mediterranean diet, bladder cancer, case-control, prevention

## Abstract

Previous studies have reported that Mediterranean diet is inversely related to the risk of several neoplasms; however, limited epidemiological data are available for bladder cancer. Thus, we examined the association between Mediterranean diet and this neoplasm in an Italian multicentric case-control study consisting of 690 bladder cancer cases and 665 controls. We assessed the adherence to the Mediterranean diet via a Mediterranean Diet Score (MDS), which represents the major characteristics of the Mediterranean diet and ranges from 0 to 9 (from minimal to maximal adherence, respectively). We derived odds ratios (ORs) of bladder cancer according to the MDS score from multiple logistic regression models, allowing for major confounding factors. The ORs of bladder cancer were 0.72 (95% confidence interval, CI, 0.54–0.98) for MDS of 4–5 and 0.66 (95% CI, 0.47–0.93) for MDS of 6–9 (*p* for trend = 0.02) compared to MDS = 0–3. Results were similar in strata of sex, age, and education, while the risk appeared somewhat lower in never-smokers and patients with pT1–pT4 bladder carcinomas. Among individual components of the MDS, we observed inverse associations for greater consumption of legumes, vegetables, and fish. In our study, which was carried out on an Italian population, the higher adherence to the Mediterranean diet was related to a lower risk of bladder cancer.

## 1. Introduction

Bladder cancer is the ninth most common neoplasm in the world, particularly in high-income countries and among men [1]. The best recognized risk factor is tobacco smoking, accounting for 30–45% of cases among men and 25–30% of cases among women in Europe and North America [2]. Other relevant risk factors for bladder cancer are occupational exposure to aromatic amines, *Schistosoma haematobium* and other chronic bladder infections, phenacetin-containing analgesics, history of metabolic syndrome, history of diabetes, and genetic predisposition [3,4,5,6,7,8,9,10].

Diet may influence bladder carcinogenesis, since many metabolites of foods are excreted through the urinary tract, such as chlorination byproducts and arsenic in drinking tap water [7]. In addition, some studies have reported inverse relationships—generally of weak to moderate magnitude—with consumption of vegetables and fruit [3,11,12] as well as some micronutrients and bioactive compounds present in plant foods, such as vitamin A, vitamin C, vitamin E, folate [3], carotenoids [13], and flavonoids [14]; however, the evidence of this is inconclusive [15]. Coffee drinking and use of artificial sweeteners have been largely investigated, but any relevant association can now be excluded [7,16].

The so-called Mediterranean diet—defined as the dietary pattern of populations bordering the Mediterranean area—is a plant-centered diet that is rich in olive oil, fruits, vegetables, legumes, and whole grain cereals [17,18]. It has been recently recognized as a sustainable lifestyle model [19] and favorably related to all-cause mortality and multiple health outcomes, including cardiovascular diseases, diabetes, and cancer, in particular of the digestive tract [20,21,22,23]. To date, only two studies have investigated the role of this dietary pattern on bladder cancer, and their findings were suggestive of an inverse weak relationship that was apparently stronger among smokers [24,25] and possibly restricted to invasive tumors [25]. However, these studies were mainly carried out outside the Mediterranean area where proper adherence to this dietary pattern is limited. We therefore further explored this issue in a Mediterranean population.

## 2. Materials and Methods

### 2.1. Participants and Data Collection

The present analyses are based on a hospital-based case–control study carried out in four Italian areas—Milan, Pordenone, Naples, and Catania—in 2003–2014 [26]. Cases consisted of 690 patients (595 men and 95 women) with incident diagnosis of urothelial carcinoma of the bladder and without previous history of other neoplasms (age range: 25–84 years, median: 67 years,), recruited in major general hospitals. Most cancer cases were histologically verified (*n* = 642, 93%). According to the 2016 World Health Organization (WHO) grading system [27], 38.8% of cases (*n* = 268) were noninvasive (i.e., TNM pTis/pTa); 27.8% were pT1 (*n* = 192) and 23.0% (*n* = 159) were muscle-invasive (i.e., pT2–pT4); 44.5% of cases (*n* = 307) were moderately or well differentiated (grading, G1–G2) and 45.2% (*n* = 312) were undifferentiated or poorly differentiated (G3–G4).

Controls consisted of 665 subjects (561 men and 104 women), identified among patients admitted to the same hospitals for various non-neoplastic acute diseases that were not associated to tobacco smoking or alcohol drinking and not related to long-term dietary changes (median age: 66 years, range: 27–84 years). Among controls, 22.1% were admitted for nontraumatic orthopedic disorders, 28.9% for traumas, 39.3% for acute surgery, and 9.8% for various other illnesses.

We submitted the study protocol to the Board of Ethics of the participating hospitals, which gave their approval, and all participants provided a written informed consent.

Participants’ information were collected during the hospital stay by trained interviewers using a structured questionnaire. More than 95% of eligible cases and controls agreed to participate. In particular, data concerning sociodemographic characteristics, anthropometric parameters, lifetime tobacco and alcohol consumption, occupational exposure to specific chemical substances, and personal medical history of selected diseases were registered. Specifically, dietary habits in the two years preceding diagnosis for cases or interview for controls was evaluated using a valid and reproducible food frequency questionnaire (FFQ), including 80 foods and recipes as well as different types of alcoholic beverages [28,29]. Subjects were requested to report their habitual frequency of consumption per week for each item. Occasional consumption, i.e., frequency greater than one per month and lower than one per week, was considered as 0.5 per week. We used an Italian food composition database to obtain estimates of nutrient and total energy intakes [30,31].

### 2.2. Mediterranean Diet Score

We evaluated the adherence to the Mediterranean diet through the score proposed by Trichopoulou et al. [32] based on nine dietary components. Operatively, the method consists of assigning a value of 0 or 1 to each score components depending on consumption of study participants. In particular, for components typical of the Mediterranean diet (namely, fruits and nuts, vegetables, legumes, cereals, fish and seafood, and high monounsaturated/saturated fatty acids ratio), a value of 1 is given to subjects with an intake greater or equal to the sex-specific median, while a value of 0 is given to participants with an intake lower than the median. Conversely, for components that are not typical of the Mediterranean diet (namely, dairy products and meats), a value of 1 is given to subjects with a consumption lower than sex-specific median, whereas a value of 0 is given to subjects with a consumption greater than the median. As for alcohol consumption, men are given a value of 1 if they consumed between 10 and 50 g of ethanol/day and 0 otherwise, while women are given a value of 1 if they consumed between 5 and 25 g of ethanol/day and 0 otherwise. We then obtained the Mediterranean Diet Score (MDS) summing the nine components’ values. Therefore, the score ranges between 0 and 9 (representing minimal and maximal adherence, respectively).

### 2.3. Statistical Analysis

We assessed the differences between cases and controls in terms of categories of the main covariates using the χ^2^ test. We derived the odd ratios (ORs)—and their 95% confidence intervals (CIs)—of bladder cancer according to the MDS (in categories 0–3, 4–5, 6–9, and for 1 point increment) and according to its individual components fitting unconditional logistic regression models, adjusting for sex, age (5-years categories), year of interview (as a continuous variable), study center, years of schooling (<7, 7–11, ≥12), body mass index (BMI, categories of <20 kg/m^2^, 20–24.9 kg/m^2^, 25–29.9 kg/m^2^ and ≥30 kg/m^2^), tobacco smoking (categories of never-smokers, ex-smokers, current smokers of <15 cigarettes/day, 15–24 cigarettes/day, or 25 cigarettes/day), nonalcoholic energy intake (based on quartiles among controls), history of cystitis, history of diabetes, and family history of bladder cancer. We selected the adjustment variables included in the models on the basis of a priori knowledge. We also estimated the ORs for 1-point increment of the score in strata of sex, age, years of schooling, tobacco smoking, and tumor characteristics. We assessed the heterogeneity across strata using likelihood ratio tests.

We estimated the proportion of bladder cancer cases that would have been avoided if all subjects were in the highest category of adherence to the Mediterranean diet (i.e., MDS: 6–9) using the population attributable fraction (PAF) according to the method proposed by Bruzzi et al. [33]. We computed the 95% CI for the PAF using the method described by Benichou and Gail [34,35]. Moreover, we estimated the PAF of shifting subjects to the lower adjacent MDS category (i.e., from 0–3 to 4–5, or 4–5 to 6–9) [36]. We carried out all the analyses with SAS 9.4 statistical software (SAS Institute, Cary, NY, USA).

## 3. Results

Among cancer cases, 86.2% were men and 63.1% were older than 65 years. Cases and controls were similar in terms of education (*p* = 0.80), while cases were more frequently current smokers (heavy smokers were 10.1% among cases and 3.5% among controls; *p* < 0.01) and reported more often a history of cystitis (*p* = 0.04) and diabetes (*p* < 0.01) compared to controls. Moreover, family history of bladder cancer (*p* = 0.05) and alcohol drinking (*p* = 0.05) tended to be more frequent in cases than controls (Table 1).

The ORs were 0.72 (95% CI, 0.54–0.98) for MDS = 4–5 and 0.66 (95% CI, 0.47–0.93) for MDS = 6–9 compared to the lowest category (MDS = 0–3; *p*-trend = 0.02). The OR for 1-point increment of MDS was 0.89 (95% CI, 0.83–0.96; Table 2). Accordingly, 12.6% (95% CI: −0.03%–28.4%) of bladder cancer cases would have been avoided if all subjects were highly adherent to the Mediterranean diet (i.e., MDS = 6–9). The expected reduction would be of 11.1% shifting subjects to the lower adjacent MDS category.

Bladder cancer risk did not show significant heterogeneity across strata of sex, age, and education (Table 3). The inverse association with MDS was slightly stronger among never-smokers (*p*-heterogeneity = 0.10) and among patients with pT1–pT4 tumors (*p*-heterogeneity = 0.09). No significant heterogeneity emerged for tumor grade (*p*-heterogeneity = 0.30). Moreover, we estimated similar ORs in sensitivity analyses that excluded subjects with history of diabetes (OR for 1 point increment in the MDS = 0.92, 95% CI 0.85–1.00) or cystitis (OR = 0.90, 95% CI 0.83–0.98).

When considering high vs. low consumption of the individual components of the MDS, we observed significant inverse associations for the intake of legumes (OR = 0.52, 95% CI, 0.40–0.69), vegetables (OR = 0.70, 95% CI, 0.53–0.92) and fish (OR = 0.68, 95% CI, 0.53–0.87); this is shown in supplementary Table S1.

## 4. Discussion

The findings from this Italian study indicate a favorable role of adherence to the Mediterranean diet on bladder cancer. Specifically, high adherence to such dietary pattern (i.e., MDS ≥6) was associated with an almost 35% reduced risk compared to low adherence (i.e., MDS of 0–3), with approximately 13% of bladder cancer cases attributable to a scanty adherence. Consistent results were observed in strata of relevant covariates; the inverse association with MDS appeared slightly, though not significantly, stronger for never-smokers and patients with pT1–pT4.

Only two other studies, both of which mainly based on non-Mediterranean populations, have previously investigated this dietary pattern on bladder cancer risk [24,25].

Findings from the European Prospective Investigation into Cancer and Nutrition (EPIC) cohort [24], which included 1425 incident bladder cancer cases, showed an inverse borderline association for high versus low adherence to the Mediterranean diet with a hazard ratio (HR) of 0.84 (95% CI: 0.69–1.03) based on a modified version of the original Trichopoulou’s score [32] (i.e., the 18-point rMED, which is based on the same nine components of the MDS, calculated as function of energy density, and divided into tertiles). Similar reduced risks were estimated for aggressive and nonaggressive tumors (i.e., non invasive vs invasive, HR: 0.88, 95% CI: 0.61–1.28 and HR: 0.78, 95% CI: 0.54–1.14, respectively), while a more pronounced and significant inverse association was observed among current smokers (HR: 0.66, 95% CI: 0.47–0.93), in particular among current heavy and long-term smokers (HR: 0.51, 95% CI: 0.31–0.86). Although it is plausible that the antioxidants highly present in the Mediterranean diet may help to prevent or reduce the detrimental effect on DNA of free radicals and oxidant produced by tobacco smoking, these findings should be interpreted with caution and they are not entirely comparable with our results since only 29% of the EPIC study population came from Mediterranean areas. In addition, the Melbourne Collaborative Cohort Study (MCCS), which included 379 incident bladder cancer cases, reported no association between MDS and overall bladder cancer (HR for the 5th versus 1st quintile of the MDS: 0.89, 95% CI, 0.62–1.26; HR for 1 SD increase in the score: 0.97, 95% CI, 0.87–1.07) or superficial cancer but an inverse association with invasive tumors (HR for the highest versus lowest quintile of the MDS: 0.67, 95% CI, 0.46–1.14; HR for 1 SD increase in the score: 0.86, 95% CI, 0.74–1.00) [25]. The association with invasive bladder cancer was apparently limited to former (HR for 1 SD increase: 0.80, 95% CI, 0.67–0.95) and current smokers (HR: 0.82, 95% CI: 0.63–1.05).

Although the present findings are not entirely comparable with that of the two abovementioned studies—mainly in consideration of the different populations (a typical Mediterranean population in our study vs. a European-wide population with less than a third of participants from southern Europe in the EPIC cohort and an Australian population with around 25% of Italian or Greek descents in the MCCS study) and the different a priori scores used to assess the adherence to the dietary pattern—epidemiological data are suggestive of a favorable role of a high adherence to the Mediterranean diet against bladder cancer risk.

The role of diet on bladder carcinogenesis may be mediated by many metabolites of foods, which are excreted through the urinary tract. Previous studies—although not all—on bladder cancer have shown a protective role of high intakes of fruit and vegetables and selected nutrients related to these foods [11,37]. A favorable effect on bladder cancer of olive oil has also been suggested [38]. In addition, inverse associations were reported with the intake of legumes and whole-grains products [39], while a direct one was observed for a proinflammatory diet [40], supporting an anti-inflammatory role of the Mediterranean diet [41]. Indeed, the Mediterranean diet has been related to lower levels of inflammatory markers (such as C-reactive protein, interleukin-6, and adiponectin) as well as to a reduction in oxidative cells stress. A possible explanation lies in some Mediterranean diet components that may decrease postprandial oxidative levels, which are responsible for acute inflammation state [41,42,43]. With reference to other dietary factors considered in the development of the MDS, no clear associations with bladder cancer were reported for fish [44], alcohol [45], milk/dairy products [46], or meat [47].

The lack of information on physical activity, a recognized protective factor for bladder cancer risk [48], is a potential limitation of the present study. Moreover, people who are physically active tend to be more prone to healthy lifestyle habits in general, including dietary ones, and this may have influenced our estimates. However, we limited possible residual confounding adjusting for BMI, which is related to both diet and physical activity. Limitations of the present study also include possible selection and information biases, as in most case-control studies. To overcome such limitations, we did not include in the control group patients with diagnoses associated to long-term dietary modifications, alcohol drinking, and tobacco smoking. In addition, we selected cases and controls from the same catchment area and obtained a participation rate of over 95%. Face-to-face interviews were conducted by trained interviewers within similar hospital settings for cases and controls to limit possible information bias. Strengths of our investigation include the evaluation of dietary habits of a southern European population where Mediterranean diet is still predominant, the use of a reproducible [29] and valid [28] FFQ, and data on several confounding factors available for adjustment purposes. However, a residual confounding effect of tobacco smoking and other lifestyle factors cannot be completely ruled out.

In conclusion, our investigation from Italy showed that an elevated adherence to the Mediterranean diet reduces bladder cancer risk.

## Figures and Tables

**Table 1:** Distribution of 690 cases of bladder cancer and 665 controls according to age, sex, education, and other selected variables. Italy, 2003–2014.

Characteristics	Cases *n* (%)	Controls *n* (%)	χ^2^ Test ^a^
Centre			
Milan	241 (34.9)	238 (35.8)	
Pordenone	242 (35.1)	250 (37.6)	
Naples	129 (18.7)	100 (15.0)	
Catania	78 (11.3)	77 (11.6)	
Sex			
Men	595 (86.2)	561 (84.4)	
Women	95 (13.8)	104 (15.6)	
Age (years)			
<60	148 (21.5)	178 (26.8)	
60–64	107 (15.5)	119 (17.9)	
65–69	164 (23.8)	147 (22.1)	
70–74	155 (22.5)	124 (18.7)	
≥75	116 (16.8)	97 (14.6)	
Education (years) ^b^			
<7	292 (42.4)	273 (41.1)	*p* = 0.80
7–11	224 (32.5)	215 (32.3)	
≥12	173 (25.1)	177 (26.6)	
Tobacco Smoking ^b^			
Never-smokers	96 (14.1)	237 (35.6)	*p* < 0.01
Ex-smokers	310 (45.5)	284 (42.7)	
Current smokers (cigarettes/day)			
<15	79 (11.6)	53 (8.0)	
15–24	127 (18.7)	68 (10.2)	
≥25	69 (10.1)	23 (3.5)	
Alcohol Drinking (drinks/week) ^b^			
<7	159 (23.1)	184 (27.7)	*p* = 0.05
7–<14	130 (18.9)	113 (17.0)	
14–<28	213 (30.9)	222 (33.4)	
≥28	187 (27.1)	145 (21.8)	
History of Diabetes			
No	578 (83.8)	608 (91.4)	*p* < 0.01
Yes	112 (16.2)	57 (8.6)	
History of Cystitis			
No	634 (91.9)	630 (94.7)	*p* = 0.04
Yes	56 (8.1)	35 (5.3)	
Family History Of Bladder Cancer ^c^			
No	667 (96.7)	654 (98.4)	*p* = 0.05
Yes	23 (3.3)	11 (1.6)	

^a^*p* < 0.05 from χ^2^ test identifies statistically significant differences. ^b^ The sum does not add up to the total because of some missing values. ^c^ Family history of bladder cancer in first-degree relatives.

**Table 2:** Odds ratios (OR) and 95% confidence intervals (CI) of bladder cancer according to Mediterranean diet score (MDS) among 690 bladder cancer cases and 665 controls. Italy, 2003–2014.

	Cases (%)	Controls (%)	OR ^a^ (95% CI)
MDS ^b^			
0–3	182 (26.4)	127 (19.1)	1 ^c^
4–5	308 (44.6)	318 (47.9)	0.72 (0.54–0.98)
6–9	200 (29.0)	219 (33.0)	0.66 (0.47–0.93)
			*p* trend = 0.02
1-point increment			0.89 (0.83–0.96)

^a^ Estim748ates from unconditional logistic regression models adjusted for age, sex, study center, year of interview, education, tobacco smoking, body mass index, non-alcohol energy intake, history of diabetes, history of cystitis, and family history of bladder cancer. ^b^ The sum does not add up to the total because of one missing value on the score. ^c^ Reference category.

**Table 3:** OR and 95% CI of bladder cancer for 1 point increment in the MDS in strata of selected factors and according to tumor invasiveness and tumor grade. Italy, 2003–2014.

	OR ^a^ (95% CI) MDS, 1-Point Increment
Age	
<70	0.90 (0.82–0.99)
≥70	0.88 (0.77–1.01)
	*p* heterogeneity = 0.89
Sex	
Men	0.89 (0.81–0.96)
Women	0.81 (0.64–1.01)
	*p* heterogeneity = 0.99
Education (years)	
<7	0.89 (0.79–1.01)
≥7	0.88 (0.79–0.97)
	*p* heterogeneity = 0.90
Smoking Habits	
Never	0.71 (0.58–0.87)
Ever, <20 cigarettes/day	0.86 (0.77–0.96)
Ever, ≥20 cigarettes/day	0.98 (0.86–1.11)
	*p* heterogeneity = 0.10
Tumor Invasiveness	
pTa/Tis	0.95 (0.86–1.05)
pT1–pT4	0.84 (0.77–0.93)
	*p* heterogeneity = 0.09
Tumor Grade	
Well or moderately differentiated	0.93 (0.84–1.02)
Poorly differentiated or undifferentiated	0.86 (0.78–0.95)
	*p* heterogeneity = 0.30

^a^ Estimates from unconditional logistic regression models adjusted for age, sex, study center, year of interview, education, tobacco smoking, body mass index, non-alcohol energy intake, history of diabetes, history of cystitis, and family history of bladder cancer, when appropriate.

## Supplementary Materials

The following are available online at http://www.mdpi.com/2072-6643/10/8/1061/s1, Table S1: Odds ratios (OR) and 95% confidence intervals (CI) of bladder cancer according to individual components of the Mediterranean diet score (MDS). Italy, 2003–2014.
