# Supplementary material for: Mediterranean Diet and Bladder Cancer Risk in Italy
Source: Nutrients. 2018 Aug 10;10(8):1061. doi: 10.3390/nu10081061 (PMC6115823; doi:10.3390/nu10081061)
Supplement: Supplementary file 1 [file nutrients-10-01061-s001.pdf]

Supplementary Material

Supplementary Table 1. Odds ratios (OR) and 95% confidence intervals (CI) of bladder cancer according to individual components of the Mediterranean diet score (MDS). Italy, 2003-2014.

	**OR[a] (95% CI) high vs low intake[b]**
MDS components	
Monounsaturated/saturated fat ratio	0.85 (0.67-1.09)
Legumes	0.52 (0.40-0.69)
Fruits and nuts	0.84 (0.65-1.07)
Vegetables	0.70 (0.53-0.92)
Cereals	1.13 (0.86-1.49)
Fish	0.68 (0.53-0.87)
Meat	0.90 (0.69-1.17)
Dairy products	0.81 (0.63-1.04)
Alcohol	
M: <10 g/day – W: <5 gr/day	1[c]
M: 10 - < 50 gr/day – W: 5 - < 25 gr/day	1.01 (0.76-1.34)
M: ≥50 g/day – W: ≥ 25 gr/day	1.20 (0.86-1.68)

[a] Estimates from unconditional logistic regression models adjusted for age, sex, study center, year of interview, education, tobacco smoking, body mass index, non-alcohol energy intake, history of diabetes, history of cystitis and family history of bladder cancer.

[b] With the exception of the alcohol component. High and low intakes were defined based on the sex-specific median consumption among controls.

[c] Reference category.
